# Developmental brain dynamics of numerical and arithmetic abilities

**DOI:** 10.1038/s41539-021-00099-3

**Published:** 2021-07-23

**Authors:** Stephan E. Vogel, Bert De Smedt

**Affiliations:** 1 Educational Neuroscience, Institute of Psychology, University of Graz, Graz, Austria; 2 Faculty of Psychology and Educational Sciences, KU Leuven, University of Leuven, Leuven, Belgium

**Keywords:** Cognitive neuroscience, Human behaviour

## Abstract

The development of numerical and arithmetic abilities constitutes a crucial cornerstone in our modern and educated societies. Difficulties to acquire these central skills can lead to severe consequences for an individual’s well-being and nation’s economy. In the present review, we describe our current broad understanding of the functional and structural brain organization that supports the development of numbers and arithmetic. The existing evidence points towards a complex interaction among multiple domain-specific (e.g., representation of quantities and number symbols) and domain-general (e.g., working memory, visual–spatial abilities) cognitive processes, as well as a dynamic integration of several brain regions into functional networks that support these processes. These networks are mainly, but not exclusively, located in regions of the frontal and parietal cortex, and the functional and structural dynamics of these networks differ as a function of age and performance level. Distinctive brain activation patterns have also been shown for children with dyscalculia, a specific learning disability in the domain of mathematics. Although our knowledge about the developmental brain dynamics of number and arithmetic has greatly improved over the past years, many questions about the interaction and the causal involvement of the abovementioned functional brain networks remain. This review provides a broad and critical overview of the known developmental processes and what is yet to be discovered.

## Introduction

Understanding the development of numerical and arithmetic abilities is highly relevant for our modern and educated societies. Research has shown that these abilities are equally important for life success as literacy^1^ and that deficits in these abilities can have severe effects on individuals’ well-being and nation’s economy^2^. Current estimates have shown that ~20% of the population in OECD countries have difficulties within mathematics, imposing great practical and occupation restrictions^3^. Around 5–7% of the population suffers from dyscalculia, a severe mathematical learning disorder^4^. In the last decades, cognitive neuroscientists have begun to investigate the brain mechanisms associated with the developmental dynamics of these foundational abilities. And although our current understanding is still limited, some key findings of the functional and structural organization of these networks are gradually emerging.

This review aims to reach a broad audience and only broadly summarizes our current knowledge about the functional and structural brain dynamics that are associated with the development of numerical (i.e., conceptual knowledge and representations about the structure and meaning of numbers) and arithmetic abilities (i.e., conceptual knowledge and representations about the manipulations of numbers, such as adding or subtracting, and their results). We start by discussing some general principles associated with the brain networks that support the development of these abilities. We next elaborate on the neurocognitive development of basic numerical and after that the neurocognitive development of arithmetic abilities. We then address how these abilities are impaired in the context of atypical development, i.e., in dyscalculia. We end this review by discussing the limitations of the existing body of evidence and by proposing some avenues to further investigate the developmental brain dynamics of these abilities. Throughout the review, we cautiously attempt to suggest some implications of these neuroscientific findings to everyday life, such as the classroom or clinical practice, acknowledging that such direct implications cannot be merely “dropped” in practice, yet require a collaborative effort between scientists and practitioners (for a discussion see ref. ^5^). Rather, our review aims to summarize what is scientifically know and what is not known, a base of reliable knowledge for teacher training and development that can aid practitioners to become critical consumers of so-called “brain-based” explanations.

## Principles of the brain networks associated with numerical and arithmetic abilities

A core insight that has emerged from the research is that the development of numerical and arithmetic abilities cannot be restricted or reduced to a single cognitive mechanism or to a single brain region^6^. The development of these abilities is complex and multidimensional, and consequently its education or remediation cannot be reduced to one single factor or intervention. Number and arithmetic incorporate multiple cognitive abilities^7^, representational dimensions^8^, and brain regions^9^. The neurocognitive networks and their associated functions interact in various complex ways to enable efficient and flexible processing of the relevant numerical and arithmetic information. The efficient working of these brain regions is further modulated by genetic factors^10,11^, age^12–15^, ability level^15–17^, task constraints^18,19^, education^20^, and other environmental factors such socio-economic status^21,22^.

**Fig. 1:**
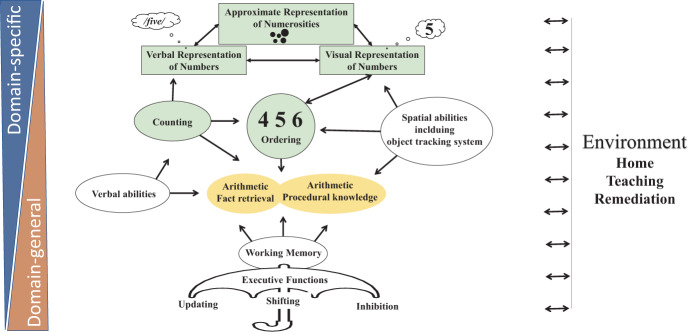
Dynamic interactions of domain-specific and domain-general functions that support the development of numerical (green shapes) and arithmetic abilities (yellow shapes). The approximate representation of numerosities, the verbal representation of numbers, and the visual representation of numbers build the core components of the triple-code model. Auxiliary functions such as verbal and spatial abilities are shown in white. The object-tracking system is part of rather domain-general visual–spatial abilities. Note that the classification of domain-specific and domain-general is rather continuous than categorical. The arrows suggest uni- or bidirectional influences.

The involved brain dynamics are, therefore, best described as an interaction of multiple brain regions/networks that vary along a functional continuum with domain-specific functions on the one end, and domain-general functions on the other end (see Fig. 1 and Fig. 3). Domain-specific functions can be defined as mental operations that are largely restricted to a particular (academic) domain. In the case of arithmetic, this involves certain aspects of basic number processing that are less relevant in other learning domains (e.g., reading). Examples include the representation of numerical quantities^23^ or symbolic knowledge about ordinal relationships^8^. Domain-general functions are less specific to a particular (academic) domain. They mainly reflect mental operations that are important for learning and information processing more generally^7^. Examples include cognitive functions such as working memory (i.e., the ability to temporally hold information in our mind) or visual–spatial reasoning (i.e., the ability to mentally manipulate and understand the spatial relation of objects), which are both relevant for learning to calculate or to read.

Another important finding from brain imaging research is that cortical brain regions are not devoted to one specific task^24^. Brain regions rather contribute to a variety of domain-specific and domain-general functions. The formation of these brain regions into functional networks is highly dynamic (see also Fig. 3). These changes can be observed in real time (e.g., the functional synchronization of different neuronal population/regions/networks depending on task requirements) as well as over larger developmental timescales (e.g., the functional specialization of neuronal populations/regions/networks to process relevant stimuli dimensions more efficiently)^25–28^. We also know that environmental factors, such as learning and education, influence the formation of domain-general and domain-specific regions into functional brain networks in various ways. For example, cognitive tutoring or specific training reconfigures the functional connectivity of relevant brain regions in elementary school^20,29,30^. The educational transition from play-based learning in kindergarten to formal learning in grade one in primary school even changes the brain activity during domain-general processes, such as executive function^20^, indicating that even these domain-general processes and their brain networks are malleable via educational practice. In the next sections, we describe the brain functions and regions that support the development of these numerical and arithmetic abilities in more detail.

## Numerical abilities

### Tuning the mind for numerical quantities

The ability to perceive and to nonverbally quantify the objects of a set (i.e., its numerosity) has been proposed as one important domain-specific precursor for the development of numerical and arithmetic abilities^31,32^. Two neurocognitive systems have been related to this development: the approximate number system and the object-tracking system^33,34^. Both systems show significant individual differences and developmental changes early in childhood. While it is undisputed that these systems provide a crucial basis for perceiving quantities, their causal relationship with the development of symbolic numerical and arithmetic abilities is debated (for reviews and detailed arguments see refs. ^35–38^).

#### The approximate number system

The approximate number system reflects an intuitive sense to estimate and to discriminate the number of objects of different sets (e.g., perceiving that a set of 16 dots differs from a set of 8 dots). In humans, this capacity increases continuously over developmental time. Six-month-old infants can reliably differentiate sets with a numerical ratio of 1:2, 9–12-month-old with a ratio of 2:3, 4–5-year-old’s with a ratio of 3:4 and adults with a ratio of 7:8 objects^31,39–43^. The approximate number system forms a central component of the triple-code model^32^ and its associated brain correlates^44^. It provides the semantic basis for two other “codes”: the visual representation of numbers, which encodes number symbols (such as the Arabic numerals), and the verbal representation of numbers, which encodes number words. The developmental formation of these codes and their corresponding brain systems in the parietal, occipital, and temporoparietal cortex have been argued to build an important foundation for the continued development of arithmetic abilities.

**Fig. 2:**
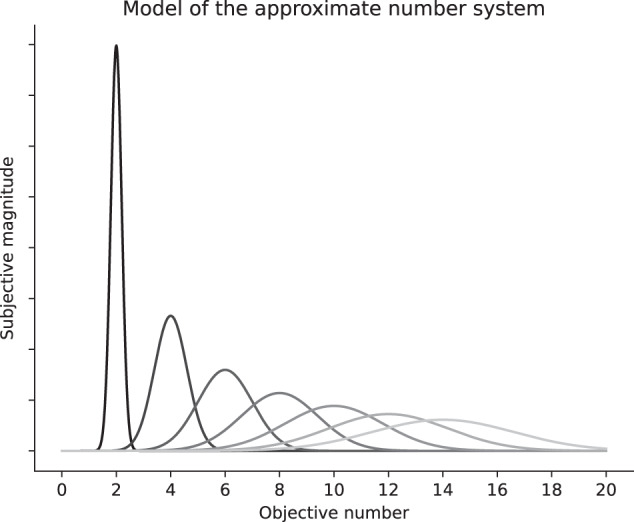
Schematic model of the approximate representation of numerical quantities. Numbers are represented as Gaussian distributions with a scalar variability (i.e., Weber’s law). The increased variability with larger numbers reflects greater uncertainty to represent these numerical quantities.

Over the last two decades, an increasing number of neuroimaging studies have investigated the neural underpinnings of the approximate number system and its development in the human brain, using a variety of different methodological approaches^45,46^. Evidence from this research indicates an early functional specialization of the parietal cortex to represent numerical quantities^23,47^. Neuroimaging studies with newborns^40^, 6-month-old infants^45,46^ as well as with young children^48,49^ have found significant numerical ratio-dependent brain activations within regions of the parietal cortex, especially the right intraparietal sulcus (IPS)^40,45,46,48,49^. Neurocognitive models of number processing propose that this approximate activation arises from a neuronal representation that encodes numbers according to Weber’s law^50,51^. More specifically, numerical quantities reflect a linear representation in which each number is represented as a Gaussian distribution with a scalar variability—the width of the distribution (i.e., tuning curves) increases with numerical quantity (see Fig. 2). Single-cell recordings in non-human primates have demonstrated that the response profiles of neurons in homolog regions of the IPS (the ventral intraparietal area, VIP) and the prefrontal cortex (PFC) behave according to this prediction—showing a systematic activation decrease as the distance to the preferred numerical quantity increases^52,53^. This work provides evidence for the existence of single neurons that encode numerical quantities within the parietal cortex of non-human primates. In humans, the neuronal tuning curves of this particular brain area are similar and their width decreases with age^12,13^, possibly indicating a developmental tuning of the neuronal populations to represent numerical quantities with a higher precision^54^. As such, the existence of the approximate number system in specific brain regions of the parietal cortex, as proposed by the triple-code model, has received great support from the neuroscientific literature (the interested reader is referred to these detailed reviews^23,31,55,56^). However, the empirical proof that the approximate number system serves as a unidirectional neurobiological foundation for the development of symbolic numerical and arithmetic abilities is rather weak^57–62^ and even non-existent^35,63,64^. Consequently, (cognitive) interventions that merely focus on training this approximate number system with the aim to transferring improvements to arithmetic have not provided conclusive evidence (for a critical review see ref. ^65^).

#### The object-tracking system

The second neurocognitive system that is implicated in the development of early perceptual abilities is the object-tracking-system^33,34^. This system enables the precise and fast individuation of 1–4 objects within a visual scene (in the literature often termed subitizing). The primary function of the object-tracking system is to perceive object boundaries, to predict object movements, and to accurately retain a small number of objects in working memory. As such, the object-tracking system is considered to be more of a domain-general system of visual–spatial abilities^31,34^. The capacity of the object-tracking system to keep track of a small number of objects develops rapidly over the first month of infants. Whereas 6-month-olds display a capacity limit of one object, 12-month-olds already show adult-like object-tracking abilities (i.e., 3–4 objects)^66^. Because of its properties to perceive a limited number of individual objects with high precision, it is thought to function as an important primitive for the conceptual development of counting via subitizing (see section 2.2.3)^67^.

Neuroimaging studies with adults have identified several brain regions that correlate with the object-tracking system. These brain regions encompass the inferior parietal cortex, the posterior parietal cortex, the occipital cortex^68–71^, and the temporoparietal junction (TPJ)^72,73^. While early neural responses of the occipital cortex seem to be driven by basic visual parameters and object identification^74,75^, the activation of the parietal cortex has been linked to enumeration. The TPJ might play a particular role in this process as its brain activation has been linked to the ventral attention network, which directs attention to salient object properties of the visual scene (i.e., the number of elements).

The developmental brain mechanisms of the object-tracking system, as well as its separation (or overlap) from the approximate number system, are still unclear. The results of a study with 6-month-old infants suggested a separation of the two systems and an involvement of both systems early in development^46^. The overall question of whether the enumeration of objects is exclusively performed by the approximate number system (i.e., the single-system view), or whether small numerosities are encoded by the object-tracking-system and large numerosities by the approximate number system (i.e., the double system view) is not yet resolved and debated (the interested reader is referred to these detailed reviews^31,34^).

### Introducing cultural tools: the role of symbolic numerical knowledge

Sophisticated numerical abilities of children go well beyond the mere perception of quantities. One important stepping stone is the construction of symbolic numerical knowledge: children have to learn to represent numerical information via symbols, which can be number words or Arabic digits. One prominent account proposes that over developmental time (and education), number symbols, which are argued to be processed in the occipital cortex (see triple-code model), are mapped onto quantities represented in the IPS^31,76^. In other words, it is argued that due to a “symbolic-quantity-mapping” an associative link between cultural invented symbols and the neurobiological foundation of quantities is established: Children associate arbitrary visual symbols (e.g., the Arabic digit “5”; or the number word */five/*) to corresponding quantities (e.g., “the five-ness”). They further learn via counting to use these symbols to determine the exact quantity of a set.

#### The symbolic representation of quantities

The neuroscientific evidence of how our human brain develops symbolic representations has substantially increased over the past years^77,78^. There is now good evidence to support the hypothesis that specific regions of the visual cortex are responsive to the visual properties of number symbols (the interested reader is referred to this meta-analysis^79^). Several studies have revealed increases in brain activity in the right and/or left occipitotemporal cortex, close to the fusiform gyrus of the inferior occipitotemporal gyrus^79–81^. The locus of this left-hemispheric activity is close to the visual word form area, which is critical to recognize letters and to read words^82^. As such, it has been suggested that activity in the left and right occipitotemporal cortices reflect the increased efficiency to process visual number symbols such as Arabic digits^83^. Although this view is consistent with the triple-code model, the specifics for functional specialization in the left and/or the right hemispheres as well as their possible lateralization to process number symbols are still unresolved.

Findings from this work have also shown that similar and distinct brain regions as in perceiving the number of objects are activated during mere symbolic number processing, especially in the parietal and frontal cortex (the interested reader is referred to this meta-analysis^84^). The overlapping brain activation is seen as one of the core findings to support the “symbolic-quantity mapping” account. It suggests that the human brain processes numerical quantities in the same brain region, independent of notation (i.e., symbolic or nonsymbolic) in which quantities are presented^85,86^. However, several findings, including that of distinct brain regions, have challenged this idea and questioned whether the observed overlap in brain activation provides conclusive evidence for such simple mapping (for a discussion, see ref. ^38^).

Specifically, neuroimaging studies were able to demonstrate that regions beyond the parietal cortex, especially the frontal cortex (the middle frontal gyrus, the inferior frontal gyrus, and the precentral gyrus), additionally engage in symbolic encoding^48,87,88^ and that symbolic processing mechanisms mediate the relation between nonsymbolic encoding and math, even after controlling for multiple domain-general functions^38^. The activation of brain regions outside the parietal cortex is substantially greater in children compared to adults^87^. This additional brain activation might be explained by auxiliary functions that support symbolic computations (e.g., working memory; executive functioning), or by a direct encoding of symbolic-quantity information in these regions^47,89^. Although both alternatives are possible, the precise answer is not yet known. These more recent neuroimaging studies have also revealed that the neuronal responses to perceiving the number of objects and to process symbolic quantities are very heterogeneous with regard to their location, even within the IPS^90–93^. In other words, although similar brain regions may encode both nonsymbolic and symbolic representations, the precise neuronal encoding patterns differ within these regions. It is important to know that these studies were done in adults and that there are no studies available that have investigated developmental changes in relation to these different encoding patterns in children, an issue that warrants further investigation.

Additional findings suggest a developmental specialization of several brain regions to process symbolic numerical information. This specialization is characterized by age and/or training/education-related increase in brain activation in the parietal cortex, the inferior frontal cortex, and the occipital cortex^14,30,94,95^, and an age and/or training/education-related decrease in brain activation in several regions of the prefrontal cortex, including the anterior cingulate gyrus^30,87,95^. This functional shift in brain activation has been related to the automatization of the parietal and occipital cortex, in particular the left IPS, to process symbolic numerical information as children get more experienced with this stimulus dimension^14,94,95^. The age and training/education-related decrease in frontal and cingulate brain regions is often associated with a reduced working memory load and less attentional effort in adults or more skilled participants compared to children^87,95^. These findings are in line with further evidence that has shown that the brain activation of the left parietal cortex is associated with behavioral skills that require the manipulation of numerals (e.g., arithmetic)^94,96^. The developmental specialization of the left IPS could therefore reflect a dynamic interaction of domain-general as well as domain-specific resources to efficiently act upon symbolic knowledge. Indeed, a recent study showed that functional connectivity patterns (i.e., the signal correlation between distant brain regions) between the right parietal and the left parietal cortex predicted individual scores in a standardized test of mathematical achievement^97^. Together, these findings indicate that the learning of symbolic representations is much more complex than simply mapping quantities onto symbols (see the following references for a detailed discussion^35–37,64^). They suggest that the construction and learning of symbolic numerical information are related to the integration of multiple knowledge dimensions^98^, such as numerical order and counting, all of which should be fostered through (mathematics) education.

#### Knowledge of numerical order

Numerical order refers to our knowledge that an Arabic numeral or number word occupies a relative rank or position within a sequence^8^. Research that aims to better understand the functional development of numerical order processing has significantly increased over the past years. Results from this work have demonstrated that the understanding of numerical order (a) explains unique variance in children’s arithmetic abilities^15,99–103^, (b) mediates the well-established association between numerical quantity processing and arithmetic^15,100,104^, and (c) shows a specific developmental trajectory within the first years of formal education, becoming the best predictor and diagnostic marker of arithmetic performance at the end of primary school^99^.

Investigations into the developmental brain mechanisms of numerical order processing are extremely sparse. Only a handful of neuroimaging studies have directly explored the neural correlates of numerical order processing in children. The results of these studies have demonstrated an age-dependent increase in brain activation in the left IPS in response to numerical order processing^15,17,30,105,106^. In addition, a significant association between the neural responses of ordinal processing and arithmetic performance in regions of the semantic control network was found, especially in the right posterior middle temporal gyrus (pMTG) and at the right inferior frontal gyrus (IFG)^15^. The IFG is known for its functional relevance in visual working memory, which helps to monitor simple rules that are associated with the manipulation of numerical items^107^. These findings indicate again that domain-general and domain-specific resources work in concert to support multiple dimensions that are involved in the construction of symbolic representations in the brain.

#### Counting: the bridge to arithmetic?

Another knowledge dimension that is critical for the construction of symbolic numerical information is the ability to count. Counting allows children to determine the exact quantity of elements in a given set by using a symbolic representation, i.e., a number word and later an Arabic numeral. Counting is also related to the understanding of order, given that counting constitutes the precise matching of an ordered sequence of symbols with quantities. The development of counting begins at around 2–3 years of age when children start to learn number words and the associated stable order principle^108,109^, i.e., knowledge that a particular number word, such as */nine/*, comes after the number word */eight/* and before the number word */ten/*, which appears to increase with age and the understanding of this principle has been shown to be delayed in children with difficulties in learning arithmetic^110^. Over the ages of 4–5 children gradually develop an understanding that number words represent the number of objects and, therefore, their numerical quantities. This insight is associated with the knowledge that each counting word needs to be lined up with only one object in the set to be counted (i.e., one-to-one correspondence principle) and that the last word reflects the total number of objects (i.e., cardinal principle)^111^. The process of counting objects is often accompanied by the use of fingers, which might facilitate the spatial representation of numbers^13,112,113^. During this time children also conceptually grasp the idea that every number in the counting list has a predecessor (*n* − 1) and a successor (*n* + 1)^114,115^. Children who have mastered these general conceptual ideas are known as cardinal principle knowers and recent evidence indicates that symbolic numerical knowledge accelerates after children have mastered these cognitive steps^116^.

Unfortunately, we know hardly anything about the brain mechanisms that facilitate this development. One reason for this lack of knowledge is that neuroscientific data collection (e.g., fMRI) is particularly difficult in children of this age range, i.e., 3–5-year olds, as the acquisition of such data requires children to lay very still in the magnet for a given amount of time. The few studies that have investigated the brain mechanisms of counting, albeit in older populations, have indicated a link to the approximate number system (see section 2.1.1), and/or to the object-tracking system (see section 2.1.2). More specifically, effortful counting of larger sets appears to be associated with brain activity in fronto-parietal regions, while the enumeration of smaller sets is linked to temporal-parietal brain regions, especially to the TPJ^75^. The involvement of the TPJ in counting indicates that the precise individuation of smaller sets of elements via the object-tracking system (i.e., subitizing) may feed into the conceptual learning of counting^67^. This is further supported by the fact that children first understand the principles of counting in the small number range. Once children have grasped these principles for smaller numbers, they can apply this knowledge to an infinite number of elements^117^. However, no brain imaging study has yet systematically mapped the progressive development of counting: from understanding that counting words always occur in the same ordered sequence (the stable order principle) to knowing that the last word in the sequence represents the total number of objects in the set (cardinality principle). This clearly represents an area for future (brain imaging) research.

## Arithmetic abilities

### Arithmetic development as a strategy change

Children’s counting is not only important for their acquisition of symbolic knowledge. It also provides a crucial foundation for their development of arithmetic. This development is characterized by a change in the distribution of strategies that children use to calculate, i.e., to determine the sum or difference of two (or more) quantities^118^. Initially, children use fingers or manipulatives to count the answer to a problem^119^, yet progressively, they execute strategies without external aids. The counting strategies become increasingly sophisticated and children move from counting all elements (sum strategy), to counting from the first (counting on), and subsequently from the larger (counting-on-larger) operand. Through practice, the strength of the problem–answer associations is increased, and eventually, this results in the storage and retrieval of arithmetic facts from long-term memory. These arithmetic facts further provide the basis for more complex procedural calculation strategies, such as decomposition strategies, in which a problem is decomposed into more simple problems, as is the case when children work with larger numbers (e.g., 8 + 6 is solved by doing 8 + 2 = 10 and 10 + 4 = 14, so 14). It is important to acknowledge that the abovementioned types of strategies all remain available over development, but that their distributions change^120^.

### The arithmetic brain network

**Fig. 3:**
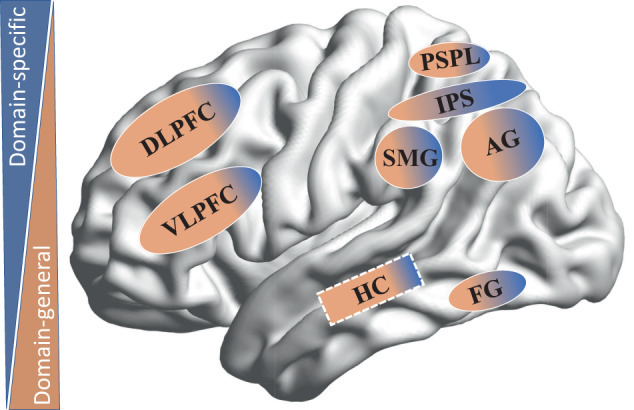
Brain regions of the arithmetic network. The blue and orange color coding indicates the relative domain-specific and domain-general involvement of the particular brain regions. DLPFC dorsolateral prefrontal cortex, VLPFC ventrolateral prefrontal cortex, PSPL posterior superior parietal lobe, IPS intraparietal sulcus, SMG supramarginal gyrus, AG angular gyrus, FG fusiform gyrus, HC hippocampus (the dotted lines indicate the medial position of this brain region).

What are the brain regions that are correlated with the development of these strategies? The nascent body of brain imaging studies has revealed a widespread set of interconnected brain areas (see Fig. 3), reflecting the involvement of both domain-specific and domain-general processes^9^. As already discussed further above, a key region within this network is the IPS. It has been suggested that the brain activation of this region reflects quantity processing. Increases in activity in this region are typically observed during the execution of procedural strategies, such as counting and decomposition strategies, which are more often observed during the solution of larger problems and during subtraction. The retrieval of arithmetic facts from long-term memory has been associated with the more inferior part of the parietal cortex, which includes the angular gyrus (AG) and the supramarginal gyrus (SMG). Activity in these regions is typically correlated with multiplication, which is known to be dependent on fact retrieval. Although studies in adults have observed increases in the AG during the retrieval of arithmetic facts, this has not always been consistently observed in children. Here, activity in the hippocampus has been shown to correlate with arithmetic fact retrieval^121,122^. This is not unexpected in view of the fact that the hippocampus plays a role in memory encoding^123^. Therefore, it has been suggested that arithmetic fact retrieval is a graded phenomenon, in which the early and initial consolidation stages are more related to activity in the hippocampus and the later more automatized stages are related to activity in the AG.

The arithmetic network also includes auxiliary areas, including the prefrontal cortex and the posterior superior parietal cortex (PSPL), related to working memory and the allocation of attentional resources. Activity in these areas typically increases with the difficulty of the task, and these increases are often observed during more complex problems or during the early stages of learning^9^. Arithmetic training studies in adults^124^ and children^30,125^ have shown that brain activity in these auxiliary areas decreases after training.

The brain activity during arithmetic undergoes important developmental changes, yet there are only but a handful of studies that have investigated these changes. One piece of evidence comes from studies that have compared children in different age groups^80^ or that have correlated brain activity with age^126,127^. These studies have generally shown that with increasing age, brain activity decreases in prefrontal areas and increases in the parietal cortex and the occipitotemporal cortex. These changes seem to mirror the development that can be observed in children’s arithmetic strategy use, which is characterized by an increasing automatization and reliance on arithmetic fact retrieval. To the best of our knowledge, there is only one study that has investigated developmental changes at multiple time points in the same sample^122^. The results showed decreases in brain activity in the prefrontal cortex as well as increases in the hippocampus. An analysis of children’s arithmetic strategy use revealed that these changes were accompanied by an increase in fact retrieval and a decrease in counting strategies.

Children show large individual differences in their development of strategies^128^ in the classroom. Are such differences also observed in the brain? A small number of studies have observed that individuals with lower mathematical skills show higher activity in the IPS during calculation^121,129–131^. The precise interpretation of this association remains, however, unclear^9^. It is possible that the increased IPS activity in children with lower mathematical skills reflects a protracted reliance on immature calculation strategies, such as counting. In contrast to their peers, poorer numerical processing skills prevent these children from developing a reliance on the arithmetic fact retrieval network (i.e., in the adjacent AG and SMG areas of the parietal cortex). This shift has to be differentiated from the developmental increase in brain activity of the IPS in response to numerical processing. While in the context of low mathematics achievement, the brain activity during arithmetic might relate to a delayed shift of strategies and the corresponding brain networks, the brain activity during numerical processing corresponds to an age-related deficiency to processes symbolic numerical quantities. As such, it is possible that individuals with lower mathematical skills show age-dependent reduced brain activation in the IPS in response to symbolic numerical quantity processing, as well as greater activation in the IPS in response to arithmetic problem-solving, due to their protracted reliance on symbolic-quantity processing instead of arithmetic fact retrieval.

Individual differences in arithmetic performance have also been correlated with structural characteristics of the arithmetic brain network, such as the anatomical structure or volume of brain areas (gray matter) as well as the connections between them (white matter), although the number of existing studies remains to be small. The evidence from this work suggests that the gray matter volume of the arithmetic network is positively correlated with higher arithmetic skills^132,133^. Two key findings have emerged from studies that have investigated white matter connections between distant brain regions and arithmetic performance^134^: (a) positive correlations between white matter tracts that connect the prefrontal cortex and the posterior parietal cortex (i.e., the superior longitudinal fasciculus and arcuate fasciculus)^135–137^, and (b) a positive association between the tract that connects the prefrontal cortex and the occipitotemporal cortex (i.e., the inferior longitudinal fasciculus)^138,139^. These structural brain imaging data indicate that larger gray matter volume and a better white matter organization of the connections between distant areas of the arithmetic network coincide with better arithmetic performance. In the absence of longitudinal data, we currently do not know whether these structural variations are the cause or consequence of arithmetic development. In other words, it is unclear whether these structural characteristics precede the individual differences in arithmetic development or whether they emerge as a result of these individual differences (and related expertise).

## Atypical development: dyscalculia

Approximately 5–7% of children experience life-long and persisting difficulties in acquiring arithmetic skills and this condition is referred to as dyscalculia^4^. The difficulties are not merely explained by sensory problems, low intellectual ability, and mental or neurological conditions^4^. For decades, it has been emphasized that difficulties in arithmetic strategy use are the hallmark of dyscalculia^140–142^. In more recent years, it has been observed that children with dyscalculia also show consistent impairments in the processing of symbolic numbers^143,144^, for which reason it has been suggested that measures of symbolic number processing might be useful diagnostic markers for children at risk for dyscalculia^145–147^. These impairments seem to be persistent over time and they coincide with arithmetic fact retrieval deficits, although it remains to be unclear whether these difficulties in number processing are the cause or the consequence of poor arithmetic development or both.

The etiology of dyscalculia is still debated and it is likely that this etiology is heterogenous^6^. Evidence indicates that genetic, neurobiological, cognitive, and environmental factors might contribute to the atypical development of brain systems that impair the representation and/or acquisition of numerical abilities (for reviews, see refs. ^140,148,149^). Our neuroscientific evidence on the origins of dyscalculia is, however, limited because longitudinal investigations are still missing (for an exception, see ref. ^106^). The existing body of cross-sectional studies is descriptive in nature, revealing insights about the phenotype but not about the origin of a disorder. Stated differently, it is currently unclear whether the structural and functional brain abnormalities in dyscalculia are the cause or the consequence of the learning disorder. In other words, are the abnormalities present before the learning disorder manifests itself (= cause)? Or do these abnormalities emerge as a result of a poor learning process or experience with mathematics (= consequence)? This question can only be answered via longitudinal data, part of which is collected at an early age before the disorder emerges, which is currently lacking at the neurobiological level. Nevertheless, domain-specific, as well as domain-general neurocognitive deficits, have been hypothesized. The predominant domain-specific accounts propose either a neurocognitive deficit^148^ to process and to represent numerical quantities or a deficit to access^150^ numerical quantities via symbolic representations (e.g., Arabic numerals). The resulting consequence is similar in both accounts: individuals with dyscalculia have difficulties to develop an accurate representation of symbolic numbers that further impairs the acquisition of arithmetic abilities. The predominant domain-general account highlights the crucial role of working memory and executive functions. Especially, difficulties to inhibit irrelevant information in working memory are considered as the main cause of poor performances in arithmetic and mathematics^140^.

What do we know about the neurobiological correlates of dyscalculia? There are only a few studies that have investigated the neurocognitive correlates of basic numerical abilities in children with dyscalculia^78,151,152^. Findings from this research suggest significant differences, often deactivation, in the functional organization of the brain regions associated with nonsymbolic and symbolic number processing, in particular the IPS^153–155^. This indicates that children with dyscalculia engage similar brain networks compared with typically developing children, yet the efficiency of the involved brain regions to process numerical information seems to be altered. Although an easy explanation of these findings might be a deficient functioning of the IPS (i.e., the core deficit hypothesis) to process numerical quantities, the overall picture seems to be far more nuanced. Studies have also found greater activation during number processing in the left IPS, the frontal cortex, and in visual areas in children with dyscalculia, probably indicating that children with dyscalculia engage additional cognitive control resources to compensate for difficulties in quantity processing^17,106,153,156^. However, the exact reasons (cause or effect) for these different activation patterns are still unknown.

Only a handful of studies have investigated brain activity during arithmetic in children with dyscalculia^9,151^. These studies have observed brain activation differences in the abovementioned arithmetic network compared to typically developing children. Again, the results are difficult to interpret as both increases and decreases in brain activation have been observed. While one study showed increases in brain activity in parietal, prefrontal, and occipitotemporal regions in children with dyscalculia during addition and subtraction^157^, another study found decreases in brain activity during addition in the prefrontal cortex, right posterior parietal, and occipitotemporal areas^158^. The existing body of the data is simply too small to draw reliable conclusions on what these activation and deactivation differences mean. Although the possibility of biomarkers for clinical diagnosis of dyscalculia has been suggested^159^, this is currently not possible and we do not have enough studies available that allow us to use these brain imaging data in clinical practice.

A small number of studies have also examined the structural characteristics of the arithmetic brain in dyscalculia. This small number of morphological studies have revealed significantly less gray matter and white matter volume in the parietal cortex^160–162^, prefrontal cortex^161^, and hippocampal areas^162^ in children with dyscalculia. One recent longitudinal study found persistent reduced gray and white matter volumes over a time span of 4 years in a widespread network of frontal, parietal, temporal, and occipital regions^163^. The reduced gray and white matter volume indicate morphological and compositional alterations of the cellular microstructure in these regions such as the degree of myelination (i.e., insulation of neuronal axons that is important for efficient information processing) or differences in the shape and size of dendritic spines^164^. Independent of the neurobiological nature of these differences, changes in cellular structure might have a significant impact on the information processing of brain regions and thus might transfer to functional connectivity measures, although we are currently far from a precise understanding of how abnormalities in brain structure relate to brain function and vice versa. Indeed, a handful of studies revealed differences in the structural^162,165^ and functional^157,159^ connections between the prefrontal and parietal areas that are involved in arithmetic. The findings from this work suggest deficient structural connections as well as a functional hyperconnectivity between relevant brain regions in children with dyscalculia. These findings indicate an atypical structural and functional network formation^165^, which possibly results in less efficient integration of information processing (which would be in line with the access deficit hypothesis). Whether structural and functional measures can be related to the same or different microstructural changes, needs to be further determined. Nevertheless, recent evidence also indicates that the observed hyperconnectivity is malleable and that it can be altered via specific numerical and arithmetic interventions^29,166^. This is a promising avenue for future interventions within this domain. Although our knowledge about the atypical development of these networks has significantly increased, the current body of data is too preliminary to draw reliable conclusions and future research is needed.

## Discussion

In this article, we have provided a succinct overview of our current understanding of how the human brain supports the development of basic numerical and arithmetic abilities. The overarching finding is that the development of these abilities constitutes a dynamic interaction or co-development of multiple domain-general and domain-specific cognitive dimensions: different cognitive functions and brain regions/networks interact depending on what dimension has to be processed and how this dimension is qualitatively (e.g., calculation vs. fact retrieval) and quantitatively (e.g., efficient access to semantic information) processed at a given point in time. The quality and quantity of domain-specific functions might moderate the involvement of domain-general function (e.g., greater engagement of working memory during calculation), and the quality and quantity of domain-general functions might in turn moderate domain-specific functions (e.g., forming associations, for example between symbolic numbers and the quantities they represent, to efficiently process semantic information). These interactions occur in real time (e.g., the functional synchronization of different neuronal populations/regions during a specific task) as well as over larger developmental timescales (e.g., the functional specialization of neuronal populations/regions/networks to process relevant stimuli dimensions more efficiently). In view of these multiple dimensions, it is imperative to study large-scale connectivity patterns between different brain regions that support these dimensions rather than their mere localization^27^. The findings from a handful of studies that have started to investigate these connectivity patterns suggest a tight coupling between the frontal and parietal areas, which appears to increase with age^80^. These findings support the notion that domain-general and domain-specific functions become integrated over developmental time to support numerical and arithmetic abilities.

Although our understanding of the neurocognitive mechanisms has substantially increased over the past years, numerous challenges and questions remain. A central limitation to our current understanding is that much of our knowledge about brain mechanisms is still related to adult studies or to cross-sectional comparisons between two age groups (often children versus adults). To understand the gradual shift of cognitive functions and their associated changes of neural networks over time, longitudinal studies and cross-sectional studies that target-specific functions at specific age periods are needed^167^.

Another methodological problem is that much of the basic numerical development happens at a time period during which brain activity is difficult to investigate with the existing imaging tools, due to technical limitations (e.g., the request to lie very still in the MRI scanner for a period of time). For example, the formation of symbolic knowledge starts in preschoolers well before children are introduced to the school system. To investigate the transition from nonsymbolic to symbolic knowledge, technological and methodological advances are needed to increase the reliability and the validity of neuroimaging data at this point in development^168^.

A critical aspect that requires further investigation is the extent to which environmental factors, such as home environment and schooling, impact on the development of numerical and arithmetic abilities. Indeed, the acquisition of numerical knowledge and arithmetic does not occur in isolation, but happens within in a wider educational context^62^. Behavioral data clearly indicate that these environmental factors predict the development of numerical and arithmetic abilities^169^, yet we do not understand how these environmental factors predict and change the abovementioned brain networks that are relevant for numerical and arithmetic abilities.

Another crucial agenda for subsequent research lies in separating causes from consequences, a problem that is particularly prominent in the understanding of dyscalculia, as well as many other neurodevelopmental disorders. As documented above, there are correlations between the functional and structural properties of the arithmetic network and individual differences in performance. These correlations are almost exclusively based on cross-sectional data, which cannot separate causes from consequences. This is further complicated by the observation that studies have typically included children of very broad age ranges during which massive maturational changes in brain development occur^170^, and these maturational changes may confound the observed association between arithmetic and brain measures. In the absence of longitudinal data, we do not know whether poor arithmetic performance occurs as a result of impairments in the arithmetic brain network, or whether poor arithmetic performance leads to deviances in this network. One way to answer this question would be to study children at genetic risk for developing difficulties in their mathematics learning. Such an approach has been successful in understanding the causes and consequences of dyslexia, showing that some brain abnormalities already exist before learning to read while others occur as a result of poor reading^171^. This work has also pointed to compensatory brain mechanisms in children who are genetically at risk for dyslexia but do not develop this learning disorder. It remains to be determined whether similar processes and compensatory mechanisms can be observed in children (at risk for) dyscalculia. The discovery of such compensatory mechanisms holds great promise for designing remedial interventions in dyscalculia that might exploit these compensatory processes.

To conclude, there is an emergent understanding of the neurocognitive mechanisms associated with the early development of basic numerical and arithmetic abilities. As can be seen from this review, the available body of brain imaging evidence leaves many questions unanswered, and much more studies are needed to better understand the dynamic integration of various neurocognitive functions to establish numerical and arithmetic knowledge across development.

## Data Availability

Data sharing not applicable to this article as no datasets were generated or analyzed during this study.
